# Seawater barium and sulfide removal improved marine habitability for the Cambrian Explosion of early animals

**DOI:** 10.1093/nsr/nwae237

**Published:** 2024-07-09

**Authors:** Wei Wei, Lin-Hui Dong, Shuhai Xiao, Yi-Bo Lin, Lingang Xu, Guang-Yi Wei, Wenzhong Wang, Lan-Lan Tian, Hai-Zhen Wei, Fang Huang

**Affiliations:** State Key Laboratory of Lithospheric and Environmental Coevolution, School of Earth and Space Sciences, University of Science and Technology of China, Hefei 230026, China; CAS Center for Excellence in Comparative Planetology, University of Science and Technology of China, Hefei 230026, China; State Key Laboratory of Lithospheric and Environmental Coevolution, School of Earth and Space Sciences, University of Science and Technology of China, Hefei 230026, China; CAS Center for Excellence in Comparative Planetology, University of Science and Technology of China, Hefei 230026, China; Department of Geosciences, Virginia Tech, Blacksbrug 24061, USA; State Key Laboratory for Mineral Deposits Research, School of Earth Sciences and Engineering, Nanjing University, Nanjing 210023, China; School of Earth Sciences and Resources, China University of Geoscience, Beijing 100083, China; State Key Laboratory for Mineral Deposits Research, School of Earth Sciences and Engineering, Nanjing University, Nanjing 210023, China; CAS Center for Excellence in Comparative Planetology, University of Science and Technology of China, Hefei 230026, China; Deep Space Exploration Lab/School of Earth and Space Sciences, University of Science and Technology of China, Hefei 230026, China; State Key Laboratory of Lithospheric and Environmental Coevolution, School of Earth and Space Sciences, University of Science and Technology of China, Hefei 230026, China; CAS Center for Excellence in Comparative Planetology, University of Science and Technology of China, Hefei 230026, China; State Key Laboratory for Mineral Deposits Research, School of Earth Sciences and Engineering, Nanjing University, Nanjing 210023, China; State Key Laboratory of Lithospheric and Environmental Coevolution, School of Earth and Space Sciences, University of Science and Technology of China, Hefei 230026, China; CAS Center for Excellence in Comparative Planetology, University of Science and Technology of China, Hefei 230026, China

**Keywords:** early Cambrian, animals radiation, sulfate availability, barite precipitation, toxic material removal

## Abstract

An increase in atmospheric *p*O_2_ has been proposed as a trigger for the Cambrian Explosion at ∼539–514 Ma but the mechanistic linkage remains unclear. To gain insights into marine habitability for the Cambrian Explosion, we analysed excess Ba contents (Ba_excess_) and isotope compositions (δ^138^Ba_excess_) of ∼521-Myr-old metalliferous black shales in South China. The δ^138^Ba_excess_ values vary within a large range and show a negative logarithmic correlation with Ba_excess_, suggesting a major (>99%) drawdown of oceanic Ba inventory via barite precipitation. Spatial variations in Ba_excess_ and δ^138^Ba_excess_ indicate that Ba removal was driven by sulfate availability that was ultimately derived from the upwelling of deep seawaters. Global oceanic oxygenation across the Ediacaran–Cambrian transition may have increased the sulfate reservoir via oxidation of sulfide and concurrently decreased the Ba reservoir by barite precipitation. The removal of both H_2_S and Ba that are deleterious to animals could have improved marine habitability for early animals.

## Teaser

The removal of toxic Ba and sulfide corresponding to the global marine oxygenation during the Ediacaran-Cambrian transition could have improved marine habitability for early animals.

## INTRODUCTION

The early Cambrian (∼539–514 Ma) witnessed a rapid increase in the taxonomic diversity [1], morphological disparity [2] and ecological complexity of early animals [3]. The cause of this evolutionary event is center stage for scientific inquiries [4]. Global oceanic oxygenation [5–7] or dynamic shelf oxygenation [8] has been proposed to be the main driver. However, the O_2_ requirement for the basic metabolism of early animals may have been satisfied since the Neoproterozoic oxygenation event [9] or even earlier [10]. Some view oceanic oxygenation as a consequence of the Cambrian Explosion rather than a cause [3] and oceanic oxygenation can interact with animal evolution via positive feedback loops. Thus, the mechanistic relationship between oceanic oxygenation and early animal evolution remains enigmatic.

A Neoproterozoic marine oxygenation event may have led to the transition of oceanic sulfur (S) species from sulfide (i.e. H_2_S, HS^–^) to sulfate (SO_4_^2–^), which would have further influenced the biogeochemical cycle of barium (Ba) [11]. Unlike other heavy metals such as uranium (U), vanadium (V) and chromium (Cr), which are thermodynamically stable in oxic seawater and tend to be scavenged by reducing agents [12], Ba likely accumulated in sulfate-deficient Proterozoic waters and behaved conservatively [11,13,14] (Fig. S1). It can be removed from seawater as barite upon making contact with sulfate-bearing seawaters [11,13]. Light Ba isotopes were preferentially enriched in barite that was ultimately preserved in sediments [15–17]. Therefore, Ba isotope compositions {expressed as δ^138^Ba_sample_ = [(^138^Ba/^134^Ba)_sample_/(^138^Ba/^134^Ba)_NIST-3104a_ – 1] × 1000‰} in sediments can be a novel proxy to trace the biogeochemical cycle of Ba [13,14,17], which is useful for exploring geobiological processes responsible for the linkage between marine environmental change and biological diversity during the early Cambrian.

**Figure 1.**
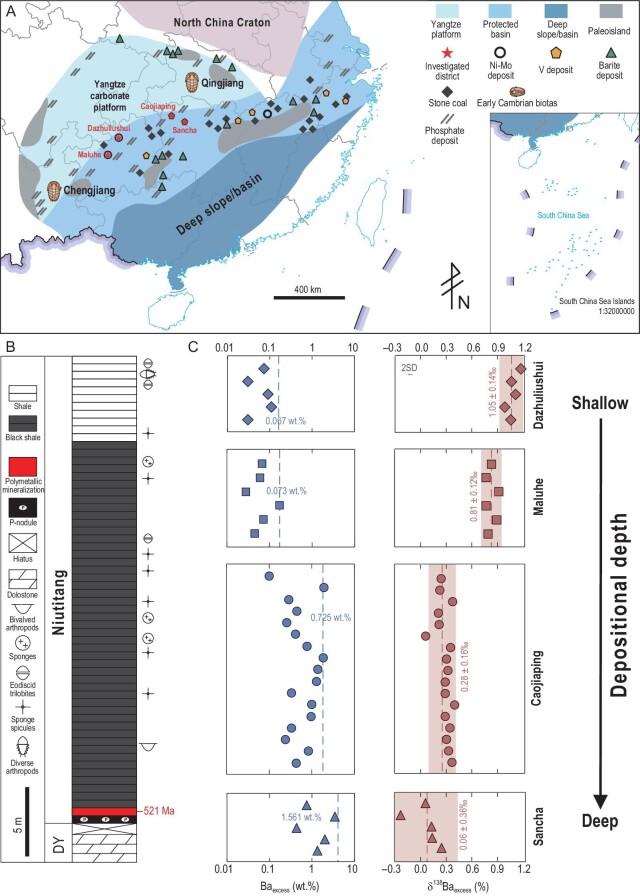
Sample localities, stratigraphic column and geochemical data. (A) Reconstructed depositional environments of the Yangtze Block during the Ediacaran–Cambrian transition. Modified after Lehmann *et al.* [20]. Stars indicate the locations of the four investigated mining districts. Trilobites indicate the locations of the early Cambrian Chengjiang and Qingjiang biotas. The distribution of the lower Cambrian stone coal, Ni–Mo–V–Ba-rich deposits and phosphorite deposits is marked. (B) Generalized stratigraphic column with the investigated stratigraphic interval highlighted. Radiometric age from Xu *et al.* [27] and Wu *et al.* [28]; biostratigraphic data from Steiner *et al.* [18]. (C) Ba_excess_ and δ^138^Ba_excess_ profiles for the metalliferous black shales from the lower Niutitang Formation at Dazhuliushui, Maluhe, Caojiaping and Sancha. In the Ba_excess_ profiles, dashed lines represent average Ba_excess_ contents. In the δ^138^Ba_excess_ profiles, dashed lines represent average δ^138^Ba_excess_ values, shades represent ±2 SD uncertainties and the error bar represents the long-term reproducibility of Ba isotope analyses of ±0.04‰. Review drawing number: GS 京(2004)1348号.

In the Yangtze Block of South China, lower Cambrian black shales are characterized by the widespread occurrence of polymetallic mineralization layers, including nickel–molybdenum (Ni–Mo), barite and V deposits as well as phosphate ore and stone coal (combustible organic-rich shale and mudstone) (Fig. 1A and B), which document remarkable fluctuations in marine chemistry [18–24]. These metalliferous black shales are slightly older than the strata that host two key Conservat Lagerstätten—the Chengjiang and Qingjiang biotas that archive the peak of the Cambrian animal radiation, and thus record paleoenvironmental conditions during the Cambrian Explosion. In this study, we analysed the contents (Ba_excess_) and isotope compositions (δ^138^Ba_excess_) of excess Ba (i.e. non-detrital Ba in sediments) of metalliferous black shales from the lower Niutitang Formation at four different mining districts in the Yangtze Block. The aim of this study is to establish the relationships between marine oxygenation, seawater sulfate increase, removal of Ba and H_2_S that are toxic to animals [25,26] and animal radiation during the early Cambrian.

## GEOLOGICAL SETTING AND SAMPLES

The lower Cambrian polymetallic mineralization layers in the Yangtze Block are hosted in the lower part of the Niutitang Formation and its equivalents [23] (Fig. 1B). The Niutitang Formation, unconformably overlying dolostones of the Ediacaran Dengying Formation or conformably overlying cherts of the Ediacaran–Cambrian Liuchapo Formation, is dominated by black shales. The lower part of the Niutitang Formation has variable lithologies, with phosphorite, barite and stone coal units at the base, overlain by centimeter-thick polymetallic Mo–Ni sulfide ores that grade laterally into V-rich shales [20]. The Niutitang Formation is fossiliferous, containing abundant sponges, arthropods and other soft-bodied and skeletal animal fossils, representing the climax of the Cambrian animal radiation [18] (Fig. 1B). A Re–Os isochron age of 521 ± 5 Ma has been reported for the polymetallic Ni–Mo unit [27] and a zircon U–Pb age of 521 ± 1 Ma has been reported for a tuff layer within the V deposits [28].

The studied samples including the Ni–Mo sulfide ores, V ores (industrial grade: V_2_O_5_ > 0.5 wt%) and host black shales were collected from the lower Niutitang Formation at the Maluhe and Dazhuliushui mining districts in Guizhou Province and Caojiaping and Sancha mining districts in Hunan Province (Fig. 1A). The Dazhuliushui mining district (27°43′13.5″N, 106°38′44.3″E) is in the western part of the Songlin dome, ∼26 km from Zunyi City; the Maluhe (26°44′40.0″N, 105°37′13.5″E) district is ∼180 km southwest of the Dazhuliushui district, near Zhijin County; the Sancha mining district (29°04′26.6″N, 110°34′32.7″E) is in Zhangjiajie City, ∼380 km northeast of the Dazhuliushui district; and the Caojiaping (29°30′9.1″N, 109°56′43.6″E) mining district is also in Zhangjiajie City, ∼80 km northwest of the Sancha district. As shown in Fig. 1A, the studied districts are located in the protected basin of the Yangtze Block [22,24], but several hundred kilometers apart. The depositional depths are relatively shallow and possibly <100 m at Maluhe and Dazhuliushui, but likely >100 m at Caojiaping and Sancha [24,29].

## RESULTS

The Ba_excess_ contents and δ^138^Ba_excess_ values of the studied samples are listed in Table S1 and are plotted in Fig. 1C. Excess Ba enrichments show an increasing trend with depositional depth, with Ba_excess_ contents of 0.031–0.109 wt% (averaging at 0.067 wt%) at Dazhuliushui, 0.028–0.171 wt% (averaging at 0.073 wt%) at Maluhe, 0.099–1.819 wt% (averaging at 0.725 wt%) at Caojiaping and 0.426–3.347 wt% (averaging at 1.561 wt%) at Sancha. The δ^138^Ba_excess_ values show an opposite trend with depositional depth, from 0.96–1.15‰ (averaging at 1.05 ± 0.14‰; 2 SD, *n* = 5) at Dazhuliushui, 0.75–0.90‰ (averaging at 0.81 ± 0.12‰; 2 SD, *n* = 6) at Maluhe, 0.06–0.39‰ (averaging at 0.28 ± 0.16‰; 2 SD, *n* = 17) at Caojiaping, to –0.24–0.24‰ (averaging at 0.06 ± 0.36‰; 2 SD, *n* = 5) at Sancha.

## DISCUSSION

### Mechanism of Ba_excess_ enrichments in the lower Cambrian black shales

**Figure 2.**
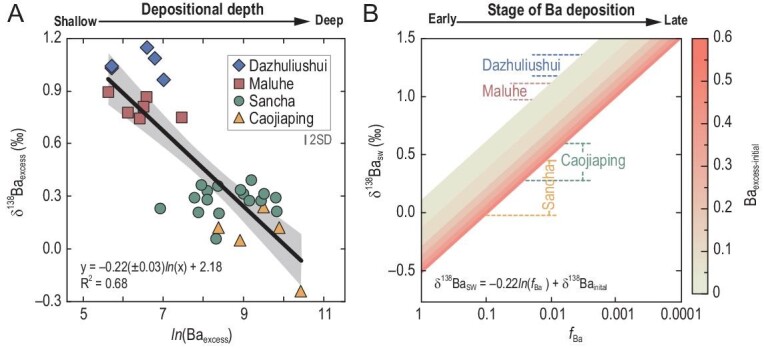
Geochemical cross-plot and sensitivity analysis. (A) Cross-plot of ln(Ba_excess_) versus δ^138^Ba_excess_ values for the metalliferous black shales from the lower Niutitang Formation at Dazhuliushui, Maluhe, Caojiaping and Sancha. Gray shade represents 95% confidence interval. Error bar represents long-term reproducibility of Ba isotope analyses of ±0.04‰. (B) Results of sensitivity analysis, showing how the seawater δ^138^Ba value (δ^138^Ba_SW_) in the protected basin of the Yangtze Block at ∼521 Ma would vary as a function of the fraction of dissolved Ba remaining in the Ba reservoir (*f*_Ba_). Calculations were conducted for the Ba_excess_ content of the initial hosting black shale (Ba_excess-initial_) from 3.347 wt% (highest value reported in this study) to 58.798 wt% (Ba content of pure barite).

Several hypotheses have been proposed for the elemental source of the lower Cambrian polymetallic mineralization layers, including a submarine hydrothermal venting origin [18,29–32], a synsedimentary seawater origin with low terrigenous sedimentation rates in an anoxic environment [19–22,24] or a combination of both [33]. Hydrothermal venting fluids are Ba-enriched relative to modern seawaters (1–119 versus 0.03–0.2 μM), while the δ^138^Ba values of the venting fluids (–0.17‰ ± 0.05‰) are lower than those of modern seawaters (0.17–0.63‰) [34,35] (Fig. S2). It seems that the negative relationship between the Ba_excess_ contents and δ^138^Ba_excess_ values (Fig. S3) could be generated by conservative mixing between hydrothermal venting fluids and seawaters. However, simply attributing this correlation to conservative mixing is problematic, because (i) the negative Ba_excess_ – δ^138^Ba_excess_ correlation is logarithmic (*R*^2^ = 0.68; Fig. 2A) rather than linear (*R*^2^ = 0.42; Fig. S3) and (ii) the elevated δ^138^Ba_excess_ values of the Dazhuliushui and Maluhe samples (0.75–1.15‰; Table S1) are much higher than those of modern marine sediments, corals, barites and even seawaters (Fig. S2). Therefore, we argue that hydrothermal activities were not solely responsible for the Ba enrichments in the lower Cambrian metalliferous black shales, although they could supply Ba.

Instead, the negative ln(Ba_excess_) – δ^138^Ba_excess_ correlation (Fig. 2A) and extremely high δ^138^Ba_excess_ values at Dazhuliushui and Maluhe (Table S1) should indicate a Rayleigh-type fractionation associated with Ba removal from the protected basin of the Yangtze Block. Based on the instantaneous equilibrium relationship, the spatial variations in the Ba_excess_ contents and δ^138^Ba_excess_ values of the metalliferous black shales can be approximated by using a Rayleigh-type distillation, as follows:


(1) \begin{eqnarray*}{{{\mathrm{\delta }}}^{138}}{\mathrm{B}}{{{\mathrm{a}}}_{{\mathrm{SW}}}} \approx {{{\mathrm{\delta }}}^{138}}{\mathrm{B}}{{{\mathrm{a}}}_{{\mathrm{initial}}}} + {\mathrm{\varepsilon }} {\rm ln} \left( {{{f}_{{\mathrm{Ba}}}}} \right)\end{eqnarray*}

(2) \begin{eqnarray*}
{{{\mathrm{\delta }}}^{138}}{\mathrm{B}}{{{\mathrm{a}}}_{{\mathrm{excess}}}} &=& {{{\mathrm{\delta }}}^{138}}{\mathrm{B}}{{{\mathrm{a}}}_{{\mathrm{SW}}}} + {\mathrm{\varepsilon }}\nonumber\\
&\approx& {{{\mathrm{\delta }}}^{138}}{\mathrm{B}}{{{\mathrm{a}}}_{{\mathrm{initial}}}} + {\mathrm{\varepsilon }}{\rm ln} \left( {{{f}_{{\mathrm{Ba}}}}} \right) + {\mathrm{\varepsilon }}
\end{eqnarray*}

where δ^138^Ba_initial_ and δ^138^Ba_SW_ are the δ^138^Ba values of the initial and remaining Ba reservoirs, respectively; ε is the Ba isotope fractionation between seawater and sediments; and *f*_Ba_ is the fraction of dissolved Ba remaining in the Ba reservoir. The Ba burial rate should be controlled by a first-order kinetic with respect to the coeval Ba reservoir and thus *f*_Ba_ is linearly scaled to Ba_excess_ in sediments [36]. Accordingly, the above equation can be simplified as Equation (3):


(3) \begin{eqnarray*}{{{\mathrm{\delta }}}^{138}}{\mathrm{B}}{{{\mathrm{a}}}_{{\mathrm{excess}}}} = {\mathrm{\varepsilon }}{\rm ln} \left( {{\mathrm{B}}{{{\mathrm{a}}}_{{\mathrm{excess}}}}} \right) + b\end{eqnarray*}

where *b* is a constant. We use this equation to fit the correlation between ln(Ba_excess_) and δ^138^Ba_excess_ (Fig. 2A), yielding an isotope fractionation of –0.22 ± 0.03‰ for Ba removal from the protected basin of the Yangtze Block.

In the marine system, oceanic Ba is scavenged primarily via barite [37], as well as carbonate minerals such as aragonite, calcite and witherite [38]. Abiogenic aragonite and calcite precipitates are preferentially enriched in heavy Ba isotopes relative to the fluids [39,40], whereas Ba isotope fractionation between aqueous fluid and witherite is minimal when the system reaches a thermodynamic equilibrium [40,41]. Biogenic carbonates are preferentially enriched in light Ba isotopes [38,42] but no biogenic carbonate component was found in the studied samples. Hence, barite precipitation may have been the main driver for the observed relationship between ln(Ba_excess_) and δ^138^Ba_excess_ (Fig. 2A), yielding a Ba isotope fractionation estimated to be –0.22 ± 0.03‰. The estimated magnitude of Ba isotope fractionation is slightly smaller than that observed in barite precipitation in laboratory experiments (from –0.35‰ to –0.25‰) [15], in modern marine systems (from –0.5‰ to –0.4‰) [17] and in modern low-sulfate lakes (–0.41 ± 0.09‰) [16], but broadly consistent with the equilibrium fractionation at 300 K from first-principle calculations (–0.23 ± 0.04‰) [40]. The larger magnitude of Ba isotope fractionation observed from laboratory experiments and natural environments, relative to the theoretical calculations, may result from kinetic (including biological) effects during the growth of barite [40]. During the extremely slow sedimentation of the metalliferous black shales in the protected basin [19–22,24], the marine Ba source was likely in isotopic equilibrium with the barite. We thus propose that enhanced barite precipitation and subsequent sedimentation were responsible for the Ba enrichments in the lower Cambrian metalliferous black shales.

### Presence of barites and potentially diagenetic effect

Scanning electron microscopy (SEM) and energy dispersive spectroscopy (EDS) analyses validate the presence of barites in the lower Cambrian metalliferous black shales at Maluhe (Fig. S4A), Sancha (Fig. S4B) and Caojiaping [11]. Barite crystals of a marine origin associated with the remineralization of organic matter (see Supplementary Materials) or marine sulfate availability are usually euhedral and ellipsoidal, with a small size of <5 μm [37]. In sediments, marine barites may be dissolved due to the complete consumption of pore-water sulfate at the methanogenesis zone and diagenetic barite fronts may form at the sulfate–methane transition zone [43,44]. This type of barite tends to be flat and tabular, with a larger crystal size of 20–700 μm [37]. The barite crystals in the studied samples show large sizes of ∼10–20 μm and irregular shapes with slight dissolution structures (Fig. S4), indicating the possible influence of diagenetic processes.

Diagenetic processes may cause a wide range of Ba isotope fractionation due to non-quantitative dissolution of barite, diffusive transport of dissolved Ba, re-precipitation of barite and pore-water leaching [13,44]. However, we argue that the diagenesis experienced by the lower Cambrian metalliferous black shales have not altered the primary Ba signals because the Ba isotope fractionation estimated from the relationship between ln(Ba_excess_) and δ^138^Ba_excess_ is fully consistent with the first-principle calculation result [40].

### Barite deposition by deep-ocean oxygenation during the early Cambrian

Barite precipitation in the marine system requires a nexus of Ba- and sulfate-bearing solutions. In the modern ocean where the sulfate supply is abundant, barite precipitation is dominated by the establishment of Ba-sufficient micro-environments via microbial remineralization of sinking organic matter (see Supplementary Materials). Thus, marine export productivity exerts first-order control on Ba accumulation in modern sediments, resulting in a positive correlation between excess Ba and organic carbon burial fluxes [45]. The observation that total organic carbon (TOC) contents are not strongly correlated with either Ba_excess_ or δ^138^Ba_excess_ (Fig. S5), however, implies that the Ba enrichments in the lower Niutitang samples were not solely controlled by export productivity, but were likely modulated by other processes, such as an increase in the oceanic sulfate concentration.

The protected basin of the Yangtze Block was prolonged and predominantly anoxic to euxinic, with an extremely low sulfate concentration during the Ediacaran and the earliest Cambrian [6,8]. In the modern Black Sea where the sulfate concentration (∼16 mM) [46] is lower than that in the open ocean (∼28 mM) [47], the dissolved Ba concentration below the O_2_/H_2_S interface is elevated to ∼500 nM due to the consumption of sulfate [48]. Thus, a replete and homogeneous Ba reservoir should have existed in the protected basin of the Yangtze Block before the Cambrian Stage 3 (521–514 Ma); marine Ba might have originated from hydrothermal vent activities in the Yangtze Block [18,29–32], as well as extraction from siliciclastic sediments by hydrothermal fluids [44,49] and redissolution of pelagic barites that formed in surface ocean [13]. This Ba reservoir should be isotopically light, considering the low δ^138^Ba values of hydrothermal fluids (–0.17‰ ± 0.05‰), upper continental crust (0.00 ± 0.04‰) and modern pelagic barites (from –0.21‰ to 0.13‰) (Fig. S2).

The sulfate source for barite precipitation in the protected basin of the Yangtze Block could have come from riverine input [8,50,51]. However, the average sulfate concentration is only ∼0.1 mM for modern river waters [52] and it was likely even lower during the early Cambrian. Such a minimal sulfate input flux cannot account for the elevated barite burial in the protected basin of the Yangtze Block [11,49]. Even if river waters could have locally higher sulfate concentrations due to the weathering of evaporites [53] and/or enhanced oxidative weathering [47,54,55], it would result in decreasing Ba_excess_ and increasing δ^138^Ba_excess_ toward offshore, which is opposite to the spatial patterns observed in the Niutitang metalliferous shales (Figs. 1C and 2A).

We propose that the sulfate source may have come from oceanic upwelling. The lower Cambrian black shales of the Niutitang Formation and its equivalents extend over ∼1600 km in the Yangtze Block, representing a widespread marine transgression [19,24], which may have widened the oceanic connection with the open ocean during the early Cambrian [6]. It is thus reasonable to infer that this transgression event may have permitted the upwelling of deep seawater to supply sufficient sulfate into the protected basin and scavenge the dissolved Ba as barite. The large sulfate reservoir in the deep ocean may be produced via the oxidation of dissolved organic sulfur, free H_2_S or sulfide in sediments by O_2_ from the atmosphere.

**Figure 3.**
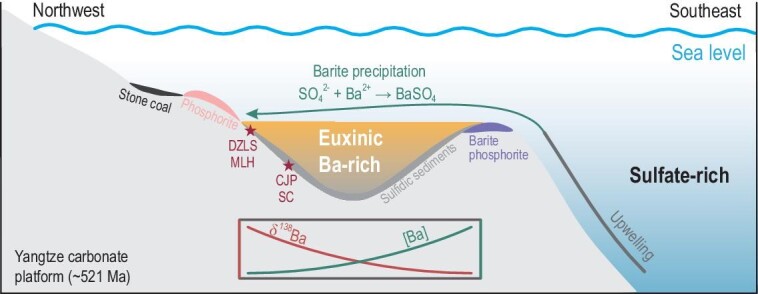
Schematic diagram showing barite accumulation in sediments and the mineralization of massive barite ores in the protected basin of the Yangtze Block induced by the upwelling of sulfate-rich deep seawater at ∼521 Ma. DZLS, Dazhuliushui; MLH, Maluhe; CJP, Caojiaping; SC, Sancha. Modified after Lehmann *et al.* [19].

Along the southeastern margin of the protected basin of the Yangtze Block, massive barite ores occur in the lowermost Niutitang Formation, beneath the polymetallic Ni–Mo–V mineralization layers [23] (Fig. 1A). Massive barite deposits are typically of hydrothermal origin in modern sulfate-sufficient oceans [56–58] but may have formed via the accumulation of hydrothermally sourced Ba in early Cambrian sulfate-limited euxinic seawater and the subsequent encounter of such Ba-rich water mass with a sulfate-bearing one [49]. Thus, the massive barite ores of the lowermost Niutitang Formation possibly represent the earliest stage of barite precipitation from the initial Ba reservoir. As the upwelled seawater migrated onshore toward the northwest of the basin, both the sulfate and Ba concentrations decreased while the δ^138^Ba value increased progressively due to the Rayleigh-type distillation related to barite precipitation (Fig. 3), leading to higher Ba_excess_ contents and lower δ^138^Ba_excess_ values in the deeper sediments (Caojiaping and Sancha) and lower Ba_excess_ contents and higher δ^138^Ba_excess_ values in the shallower sediments (Dazhuliushui and Maluhe). Thus, the spatial patterns of the Ba_excess_ and δ^138^Ba_excess_ (Figs 1C and 2A) suggest deep-ocean oxygenation at ∼521 Ma. This is also supported by lighter sulfur isotope compositions of the contemporaneous successions in the deeper basin than those in the shallow shelf of the Yangtze Block [6] and is in a good agreement with other geochemical evidence for global marine oxygenation [5–7].

As discussed above, the Ba concentration would decrease while the δ^138^Ba value of the remaining Ba reservoir in the protected basin of the Yangtze Block would increase with the progressive development of Ba deposition as barites, as expressed by the equation δ^138^Ba_SW_ ≈ δ^138^Ba_initial_ + ε ln(*f*_Ba_) [Equation (1)]. The δ^138^Ba_initial_ value can be obtained based on the Ba_excess_ content (Ba_excess-initial_) and δ^138^Ba_excess_ value (δ^138^Ba_excess-initial_) of the initial hosting black shale, which should fit the correlation between ln(Ba_excess_) and δ^138^Ba_excess_ of the studied samples (Fig. 2A), following Equation (4):


(4) \begin{eqnarray*}
{{{\mathrm{\delta }}}^{138}}{\mathrm{B}}{{{\mathrm{a}}}_{{\mathrm{initial}}}} &=& {{{\mathrm{\delta }}}^{138}}{\mathrm{B}}{{{\mathrm{a}}}_{{\mathrm{excess \hbox{-} initial}}}}-{\mathrm{\varepsilon }}\nonumber\\
&=& -0.22 {\rm ln} \left( {{\mathrm{B}}{{{\mathrm{a}}}_{{\mathrm{excess}}} \hbox{-} {{{\rm initial}}}}} \right) + 2.18-{\mathrm{\varepsilon }}\nonumber\\
\end{eqnarray*}

where ε has been estimated to be –0.22‰, Ba_excess-initial_ is supposed to be between 3.347 wt% (highest Ba_excess_ content reported in this study; Table S1) and 58.798 wt% (Ba content of pure barite) and the δ^138^Ba_excess-initial_ value is accordingly between –0.52‰ and 0.11‰ [δ^138^Ba_excess-initial_ = –0.22 ln(Ba_excess-initial_) + 2.18]. Despite the uncertainties of Ba_excess-initial_ and δ^138^Ba_initial_, the highest δ^138^Ba_excess_ value reported in this study (1.15‰; Table S1) suggests that the majority (>99%; Fig. 2B) of the replete and homogeneous Ba reservoir in the protected basin of the Yangtze Block had been scavenged at ∼521 Ma.

### Marine oxygenation, Ba removal and Cambrian radiation of animals

**Figure 4.**
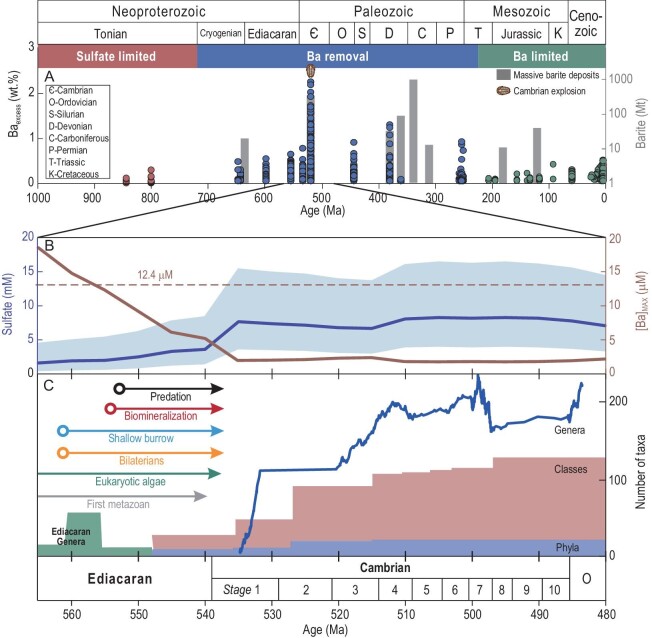
Barium removal and the Cambrian Explosion. (A) Temporal trends in Ba_excess_ contents in organic-rich mudrocks (circles) and sizes of massive barite deposits (bars) from the Neoproterozoic to the present. Modified after Wei *et al.* [11] and Han *et al.* [49], respectively. (B) Estimated mean seawater sulfate concentrations and upper limit of mean seawater Ba concentrations from 565 to 480 Ma. Sulfate concentrations from Algeo *et al.* [47], with the line representing the mean trend and shade representing the ±SD uncertainty. The dashed line represents a dissolved Ba concentration of 12.4 μM, which can lead to chronic immobilization of the crustacean animal *Ceriodaphnia dubia*, an international benchmark toxicity test species [26]. (C) Key evolutionary innovations and marine animal diversity from 565 to 480 Ma. Number of Ediacaran genera, marine animal phyla and marine animal classes from Erwin *et al.* [4]. Marine animal genus diversity from a recent analysis of the Geobiodiversity Database, which is largely based on data from China [64].

The removal of seawater Ba via barite precipitation in response to seawater sulfate increase should not be restricted to the protected basin of the Yangtze Block during the early Cambrian as described above. Based on a compilation of Ba_excess_ contents in marine organic-rich mudrocks, Wei *et al.* [11] proposed three stages of long-term changes in the oceanic Ba cycle throughout Earth history on a global scale (Fig. 4A): (i) before the late Neoproterozoic, the lack of excess Ba enrichment in sediments reflects that a large reservoir of dissolved Ba may have accumulated in the oxygen- and sulfate-deficient ocean; (ii) from the late Ediacaran to Paleozoic, enhanced Ba_excess_ enrichments in sediments and the deposition of massive barite ores [49] suggest that the accumulated seawater Ba reservoir was progressively removed due to increases in the marine sulfate reservoir and oxygenation level; and (iii) since the Mesozoic, the biogeochemical Ba cycle was mainly controlled by biological productivity in sulfate-sufficient oceans.

Across the Ediacaran–Cambrian transition (∼565–515 Ma), the global oceanic oxygenation [5–7] may have increased the mean marine sulfate concentration from 1.6 to 7.7 mM (the lower limit: from 0.4 to 3.6 mM; the upper limit: from 4.6 to 15.6 mM; Table S2) [47] and concurrently decreased the mean seawater Ba concentration. The ocean was likely saturated with respect to barite on a global scale during this period, considering that the accumulated Ba reservoir may have existed throughout the Paleozoic. On this basis, we can constrain average marine Ba concentrations based on the marine sulfate concentrations and thermodynamic solubility product of barite (Kd):


(5) \begin{eqnarray*}
{\mathrm{Kd}}\left( {{\mathrm{T}},\,{\mathrm{P}}} \right) &=& \left( {{{{\mathrm{\gamma }}}_{{\mathrm{Ba}}}} \times {{F}_{{\mathrm{Ba}}}} \times {{{\mathrm{m}}}_{{\mathrm{Ba}}}}} \right)\nonumber\\
&& \times ({{{\mathrm{\gamma }}}_{{\mathrm{sulfate}}}}\! \times\! {{F}_{{\mathrm{sulfate}}}}\! \times\! {{{\mathrm{m}}}_{{\mathrm{sulfate}}}})
\end{eqnarray*}

where Kd(T, P) is controlled by temperature and pressure, γ_i_ is the simple activity coefficient, *F*_i_ is the fraction of ions that are actually unassociated and free, and m_i_ is the product of the total molality. Assuming that the Ediacaran–Cambrian temperature and pressure were similar to the modern values (25°C and one atm), the values of Kd, γ_Ba_, *F*_Ba_, γ_sulfate_ and *F*_sulfate_ are set to be 1.1 × 10^–10^, 0.24, 0.93, 0.17 and 0.39 [59]. Accordingly, the upper limit of the mean seawater Ba concentration may have decreased from ∼18.6 to ∼2.1 μM across the Ediacaran–Cambrian transition (Fig. 4B and Table S2).

Soluble Ba compounds are toxic to marine animals [25,26] (see Supplementary Materials), although the Ba tolerance of early Cambrian animals is highly uncertain. Therefore, although the atmospheric oxygen level may have reached the threshold to support the physiology of early animals (possibly as low as 0.5%–4.0% of the present atmospheric levels) [60] before their ecological rise, early Ediacaran animals such as those in the Lantian and Weng'an biotas were likely limited to locally well-oxygenated marine environments [8,61,62] where toxic Ba was kept at low levels. From the late Ediacaran to early Cambrian, the progressive oxygenation of deep oceans [5–7], possibly as a consequence of the geobiological feedback of marine eukaryotes and animals [3,63], could have increased the sulfate reservoir via the oxidation of sulfide and concurrently decreased the Ba reservoir by barite precipitation. It is intriguing that the upper limit of the estimated mean seawater Ba concentration at ∼555 Ma was just below 12.4 μM (Fig. 4B and Table S2), which is a critical threshold that has been experimentally shown to immobilize aquatic animals [26]. Afterwards, animal diversity rapidly increased and genus-level diversity reached the first peak at the onset of the Cambrian Stage 3 [4,64] (Fig. 4C). Thus, it is plausible that the removal of toxic Ba and H_2_S corresponding to marine oxygenation may have ultimately expanded the habitable area and paved the road for the further radiation of early animals. Such positive feedback is a tenet of the Cambrian radiation and highlights the complex relationship between biological and environmental evolutions.

## CONCLUSION

We report Ba_excess_ contents and δ^138^Ba_excess_ values of the metalliferous black shales from the lower Niutitang Formation (∼521 Myr old) at four mining districts in the protected basin of the Yangtze Block. The negative correlation between ln(Ba_excess_) and δ^138^Ba_excess_ suggests that barite precipitation was responsible for the excess Ba enrichments in these shales. In addition, the spatial variations in the Ba_excess_ and δ^138^Ba_excess_ values indicate that barite precipitation was driven by sulfate availability ultimately derived from the upwelling of deep seawaters. The extremely high δ^138^Ba_excess_ values reflect that the majority (>99%) of the replete and homogeneous Ba reservoir in the protected basin of the Yangtze Block had been scavenged at ∼521 Ma. Global oceanic oxygenation across the Ediacaran–Cambrian transition may have increased the sulfate reservoir via the oxidation of sulfide and concurrently decrease the Ba reservoir by barite precipitation. The removal of both H_2_S and Ba that are deleterious to animals may have improved marine habitability for early animals.

## METHODS

### SEM and EDS analyses

Thin sections of the representative metalliferous black shale samples from Maluhe (MLH-1l) and Sancha (SC-2) were prepared for SEM and EDS analyses. The morphology and chemical composition of minerals of interest were analysed using a FEI Sirion 200 SEM equipped with a EDAX GENESIS APEX Apollo System EDS at the CAS Key Laboratory of Crust–Mantle Materials and Environments at the University of Science and Technology of China.

### Major and trace elements and TOC analyses

Contents of major elements, trace elements and TOC of the Caojiaping samples were measured using a Thermo-Fisher 6300 inductively coupled plasma optical emission spectrometry, a Thermo-Fisher Element XR inductively coupled plasma mass spectrometry and a Thermo-Fisher element analyser, respectively at Nanjing University. The associated geochemical data of the Dazhuliushui, Maluhe and Sancha samples are from Xu *et al.* [21,22] and Lehmann *et al.* [20].

### Ba isotope analyses

Chemical purification of Ba from matrix elements via ion chromatographic procedures and high-precision δ^138^Ba measurements by multiple collector inductively coupled plasma mass spectrometry were carried out at the CAS Key Laboratory of Crust–Mantle Materials and Environments. Detailed procedures are described in the Supplementary Materials.

The data quality was rigorously monitored using numbers of unprocessed pure Ba standards, processed rock reference materials and duplicated samples. The final δ^138^Ba values of the rock reference materials and samples were reported as the average values plus/minus two standard deviation (2 SD) of repeated analyses (twice) after double-spike deconvolution (Table S1). All the reported δ^138^Ba values have 2 SD better than ±0.05‰. The analysed δ^138^Ba values of the rock reference materials, SGR-1 and BHVO-2, were 0.16 ± 0.01‰ and 0.01 ± 0.00‰, consistently with the previously published values of 0.15 ± 0.04‰ and 0.02 ± 0.03‰, respectively. Moreover, the δ^138^Ba values of DZLS-17 and CJP-15 are consistent with those of the duplicates with different digestions. The long-term (>6 years) external reproducibility of δ^138^Ba values for unprocessed double-spiked pure Ba standards (SRM3104a, USTC-Ba and ICPUS-Ba) at comparable signal intensities is better than ±0.04‰ (2 SD).

### Ba correction

The samples contain variable proportions of detrital aluminosilicate components, as indicated by the large range of Al contents (0.2–7.9 wt%; Table S1). The following equations were used to obtain Ba_excess_ and δ^138^Ba_excess_:


(6) \begin{eqnarray*}
{{f}_{\mathrm{excess}}} = 1-{{\left( {{\mathrm{Ba}}/{\mathrm{Al}}} \right)}_{{\mathrm{det}}}} \times {{\left( {{\mathrm{Al}}/{\mathrm{Ba}}} \right)}_{{\mathrm{sample}}}}\end{eqnarray*}

(7) \begin{eqnarray*}
{\mathrm{B}}{{{\mathrm{a}}}_{{\mathrm{excess}}}} = {\mathrm{B}}{{{\mathrm{a}}}_{{\mathrm{sample}}}}-{{\left( {{\mathrm{Ba}}/{\mathrm{Al}}} \right)}_{{\mathrm{det}}}} \times {\mathrm{A}}{{{\mathrm{l}}}_{{\mathrm{sample}}}}\end{eqnarray*}

(8) \begin{eqnarray*}
{{{\mathrm{\delta }}}^{138}}{\mathrm{B}}{{{\mathrm{a}}}_{{\mathrm{excess}}}} &=& [ {{{\mathrm{\delta }}}^{138}}{\mathrm{B}}{{{\mathrm{a}}}_{{\mathrm{sample}}}} -{{{\mathrm{\delta }}}^{138}}{\mathrm{B}}{{{\mathrm{a}}}_{{\mathrm{det}}}}\nonumber\\
&& \times \left( {1-{{f}_{{\mathrm{excess}}}}} \right)]/{{f}_{{\mathrm{excess}}}}\end{eqnarray*}

in which the δ^138^Ba_det_ is set to be 0.00‰ based on the average δ^138^Ba value of the upper continental crust [65] and the (Ba/Al)_det_ is suggested to be 0.0037 wt%/wt% [14].

## Supplementary Material

nwae237_Supplemental_Files
