# Supplementary material for: Seawater barium and sulfide removal improved marine habitability for the Cambrian Explosion of early animals
Source: Natl Sci Rev. 2024 Jul 9;11(8):nwae237. doi: 10.1093/nsr/nwae237 (PMC11350977; doi:10.1093/nsr/nwae237)
Supplement: nwae237_Supplemental_Files [file nwae237_supplemental_files.zip › Supplementary File.docx]

Supplementary materials for

**Seawater barium and sulfide removal improved marine habitability for the Cambrian Explosion of early animals**

Wei Wei, Lin-Hui Dong, Shuhai Xiao, Yi-Bo Lin, Lingang Xu, Guang-Yi Wei, Wenzhong Wang, Lan-Lan Tian, Hai-Zhen Wei, Fang Huang

Corresponding author: E-mail: wwei1@ustc.edu.cn (W. Wei); fhuang@ustc.edu.cn (F. Huang)

**Supplementary materials include:**

Sample digestion, Ba purification, and MC-ICP-MS measurement

Modern biogeochemical cycle of Ba

Toxic effect of dissolved Ba on animals

Figures S1 to S5

References

**Supplementary data for this manuscript include the following:**

Table S1 to S2

Sample digestion, Ba purification, and MC-ICP-MS measurement

Sample powders containing ~10 μg of Ba (0.1–6 mg in sample size) were ashed for ~12 h at 600 ℃ to remove organic phases. Ashed samples were then completely digested using *aqua regia* and HF mixtures in closed Teflon beakers at 120 °C. All sample solutions were dried down and re-dissolved in 1 mL of 3 M HCl for the following cation chromatography.

The columns loaded with 2 mL of AG 50W x 8 cation resin (200–400 mesh) were rinsed by 4 M HNO_3_ + 0.5 M HF and 6 M HCl, and then were pre-conditioned by 3 M HCl. Afterwards, the samples in 1 mL of 3 M HCl and additional 28 mL of 3 M HCl were loaded to the columns, to elute matrix elements such as Ca, Mg, Al, and Sr. Barium was subsequently collected with 15 ml of 3 M HNO_3_. The collected solutions were dried down at 120 ^°^C overnight and processed with the second and similar cation chromatographic separation, in order to completely separate Ba from matrix elements. Purified Ba solutions were subsequently dried down and dissolved into 100 ppb solutions with 2% (m/m) HNO_3_ for δ^138^Ba measurements by MC-ICP-MS. The overall chemical procedure yields of Ba were >99%, and the total procedural blanks were ~1.5 ng, which could be negligible compared to ~10 μg of Ba separated from the samples.

Barium isotope compositions were analyzed using a Thermo-Fisher Scientific Neptune-Plus MC-ICP-MS. As the yields of Ba nearly reach 100%, no Ba isotope fractionation would occur during the ion chromatographic procedures. Therefore, the double-spike was added after chemical purification, in order to correct the instrumental mass discrimination. A ^135^Ba-^136^Ba double-spike with optimum ratio of 1.72 (m/m) was added into the sample solutions, with an attempt to keep ^135^Ba/^134^Ba around 23. An Aridus II desolvator (CETAC Technologies) system was adopted to introduce sample solutions. Five Ba beams (^134^Ba, ^135^Ba, ^136^Ba, ^137^Ba, and ^138^Ba) and the potentially interfering beams ^131^Xe and ^140^Ce, were simultaneously collected on Faraday cups. Masses 131 and 140 were measured to monitor interferences of ^134^Xe on ^134^Ba and ^136^Xe on ^136^Ba, and ^136^Ce on ^136^Ba and ^138^Ce on ^138^Ba, respectively. One run consisted of 60 cycles with an integration time of 2.097 seconds per cycle. The typical sensitivity of ^138^Ba was ~300 V/ppm under a low resolution mode. The background signal of ^138^Ba in 2% (m/m) HNO_3_ was <20 mV, which was negligible relative to the sample and standard ^138^Ba signals (~30 V in 100 ppb solutions).

Modern biogeochemical cycle of Ba

In soils and silicate rocks, Ba exists commonly in feldspars and micas as isomorphic replacement of K, or in Ba-rich minerals such as barite and witherite. The average δ^138^Ba value of the upper continental crust is 0.00 ± 0.04‰ [65] (Fig. S3). During continental weathering, the heavy isotopes of Ba are preferentially released [66], and subsequently delivered into the ocean via rivers [67]. The average δ^138^Ba value of the riverine input is ~0.17‰ [14,67,68]. Submarine groundwater discharge and hydrothermal venting fluids also contribute considerable Ba fluxes to the ocean, with δ^138^Ba values of ~0.12‰ [69] and –0.17‰ ± 0.05‰ [34,35], respectively (Fig. S3).

In the modern ocean, Ba has a residence time of ~11 kyr [70], not much longer than the mixing time of the ocean (~1,600 yr), behaving non-conservatively. Barium concentration and isotopic composition display quasi-nutrient type distribution, where the upper oceans are depleted in Ba and enriched in heavy Ba isotopes, and the Ba concentration increases, while the δ^138^Ba value decreases with depth [17,71–74]. This distribution of Ba concentration and δ^138^Ba value results from (1) the formation of pelagic barite in micro-environments via microbial remineralization of sinking organic matter, in which Ba is initially bonded to an amorphous P-rich phase and subsequently evolves into the barite crystal [75,76], although the majority of the modern ocean is undersaturated with respect to barite [77], and (2) partial dissolution of barite particles at depth and/or at sediment-water interface. The Ba isotope fractionation during pelagic barite precipitation (△^138^Ba_part-diss_ = δ^138^Ba_particulate_ – δ^138^Ba_dissolved_) has been estimated to be from –0.5‰ to –0.4‰ [16,17,74], while barite dissolution may not be accompanied with isotopic fractionation [16,17]. Moreover, changes to oceanic circulation (water mixing and advection) could redistribute the signals of seawater Ba concentration and isotope composition [71,73]. These processes ultimately lead to the negative correlation between Ba concentrations and δ^138^Ba values of seawaters in the global oceans [71,73,74].

Toxic effect of dissolved Ba on animals

Expanded industrial Ba uses have caused an increased awareness of environmental Ba exposure and its health impact on animals including humans [78]. It has been widely found that the Ba exposure can result in diseases of cardiovascular, renal, respiratory, hematological, nervous, endocrine, hepatobiliary, reproductive systems of animals.

In the modern ocean, although the distribution of dissolved Ba is tightly associated with biological productivity [16,45,71], high oceanic Ba concentrations are known to also have harmful effects on aquatic organisms [25,26]. Lira *et al.* [25] conducted a standardized laboratory experiments to investigate the effect of different Ba concentrations on the population development of the marine animal *Rhabditis (Pellioditis) marina*, which is a bacterivorous nematode with a very short generation time (less than 3 days) and a broad salinity and temperature tolerance [79], and is a suitable model to determine critical loads of various toxicants. At nominal Ba concentrations below 2.9 mM (measured concentrations below 365 nM due to the reaction of Ba with sulfate in the artificial seawater), Ba has no effects on the fitness and population development of *R. (P.) marina*; at nominal Ba concentrations of 14.6 mM and 26.3 mM (measured concentrations of <365 nM and 1.6 mM, respectively), the population abundance decreased; at nominal Ba concentrations higher than 35.0 mM (measured concentrations higher than 9.4 mM), the mortality increased. Due to the precipitation of barite in the artificial seawater [25], it is not straightforward to convert these experimental data to the Ba tolerance of freshwater or marine aquatic organisms.

Golding *et al.* [26] conducted an experiment to assess the toxicity of dissolved Ba to the tropical freshwater alga *Chlorella* sp. and the temperate water flea *Ceriodaphnia dubia* (an international benchmark toxicity text species) without the confounding effects from barite precipitation. *Ceriodaphnia dubia* was more sensitive to dissolved Ba than *Chlorella* sp.: A dissolved Ba concentration of 292 μM can lead to chronic immobilization of *Chlorella* sp., whereas a dissolved Ba concentration of 12.4 μM chronically inhibits the growth rate of *Ceriodaphnia dubia*.

In addition, high Ba levels in environments also have deleterious effects on other animals [25]. For example, (1) high Ba concentrations up to 36.5 mM could cause complete mortality of the oligochaete *Enchytraeus crypticus*; (2) Ba concentrations above 3.6 mM would inhibit the reproduction of the earthworm *Eisenia foetida*; (3) Ba concentrations between 73.0 μM and 730 μM caused behavioral and lethal effects on daphnids and crayfish; and (4) Ba concentrations from 1.46 μM to 4.38 μM can have adverse developmental effects on the bivalve *Mytilus californianus*.


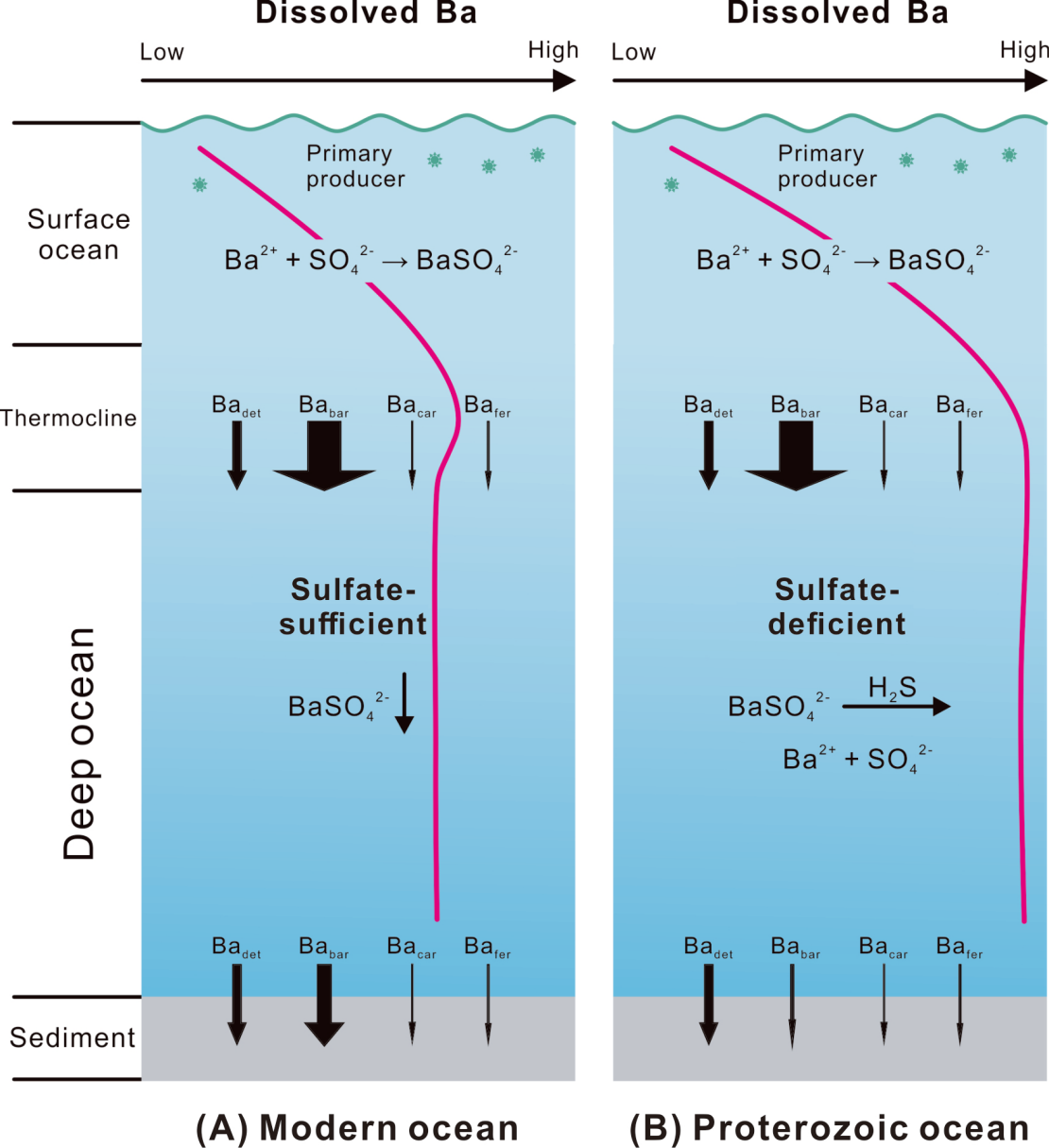


Fig. S1.

**Schematic diagram showing the biogeochemical cycle of Ba in the modern and Proterozoic oceans.** (A) Modern ocean where Ba behaves non-conservatively, primarily controlled by the formation of pelagic barite via microbial remineralization of organic matter. (B) Proterozoic ocean where Ba accumulated in sulfate-deficient seawaters and behaved conservatively. Modified after Schoepfer *et al.* [80].


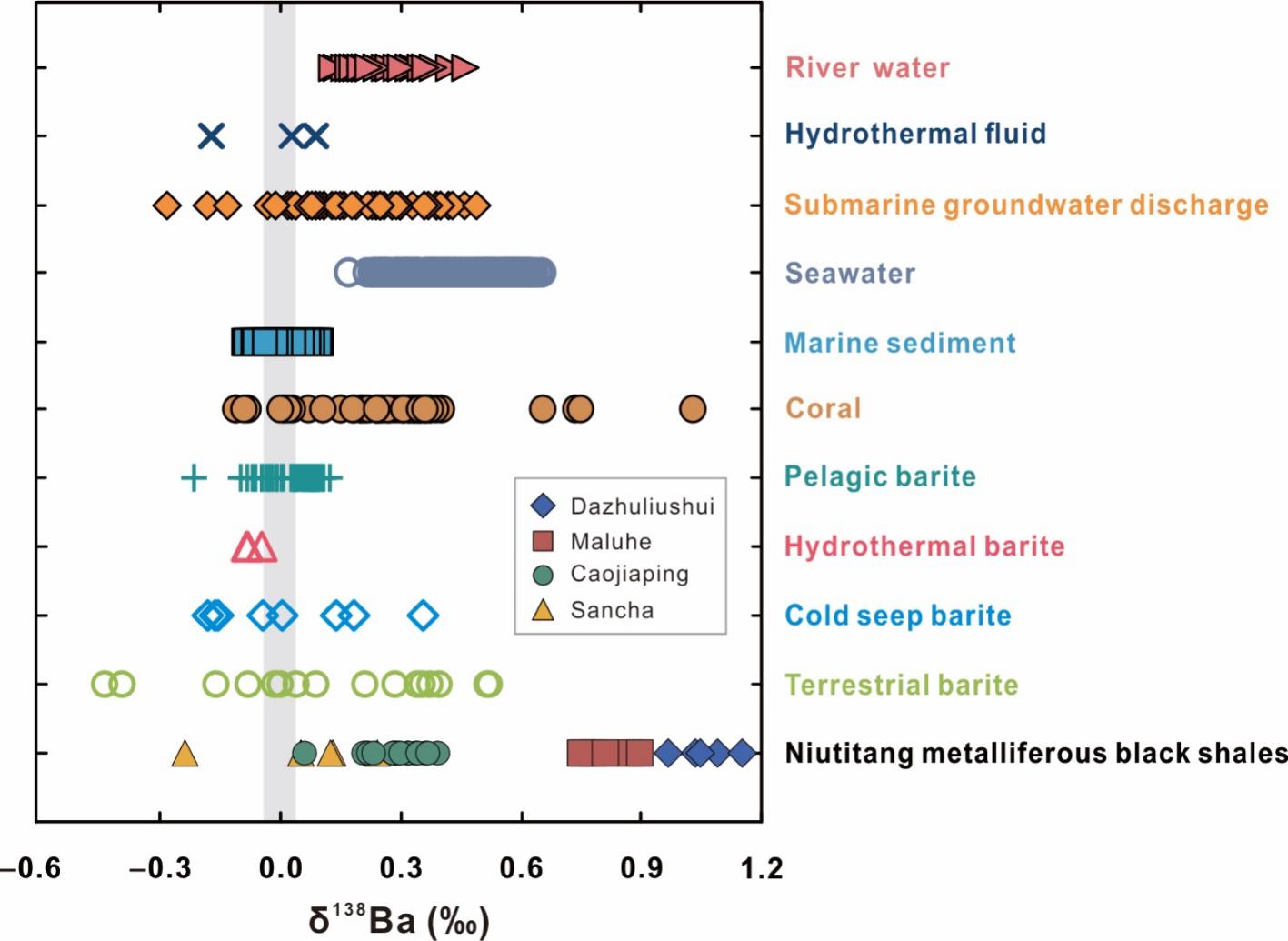


Fig. S2.

**Compilation of δ^138^Ba values of various natural materials from the literature, as well as the δ^138^Ba_excess_ values for the metalliferous black shales of the lower Niutitang Formation at Dazhuliushui, Maluhe, Caojiaping, and Sancha**. Data are sourced from Cao *et al.* [68] and Gou *et al.* [67] for river waters, from Hsieh *et al.* [34] and Zhang *et al.* [35] for hydrothermal fluids, from Mayfield *et al.* [69] for submarine groundwater discharge, from Horner *et al.* [71], Pretet *et al.* [81], Bates *et al.* [73], Hsieh and Henderson [74], Bridgestock *et al.* [17], Hemsing *et al.* [82], Geyman *et al.* [42], and Cao *et al.* [68,83] for seawaters, from Bridgestock *et al.* [17] for marine sediments, from Pretet *et al.* [81], Hemsing *et al.* [82], Liu *et al.* [38], and Geyman *et al.* [42] for natural corals, and from von Allmen *et al.* [15] and Crockford *et al.* [13] for natural barites. The grey vertical bar represents the average δ^138^Ba value of the upper continental crust (0.00 ± 0.04‰) [65].


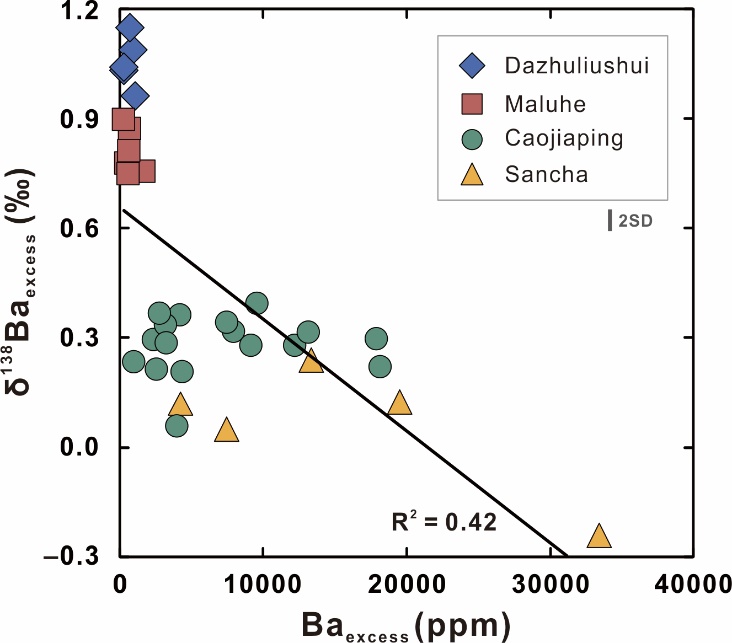


Fig. S3.

**Cross-plot of Ba_excess_ contents versus δ^138^Ba_excess_ values for metalliferous black shales from the lower Niutitang Formation at Dazhuliushui, Maluhe, Caojiaping, and Sancha.** Error bar represents long-term reproducibility of Ba isotope analyses of ±0.05‰.


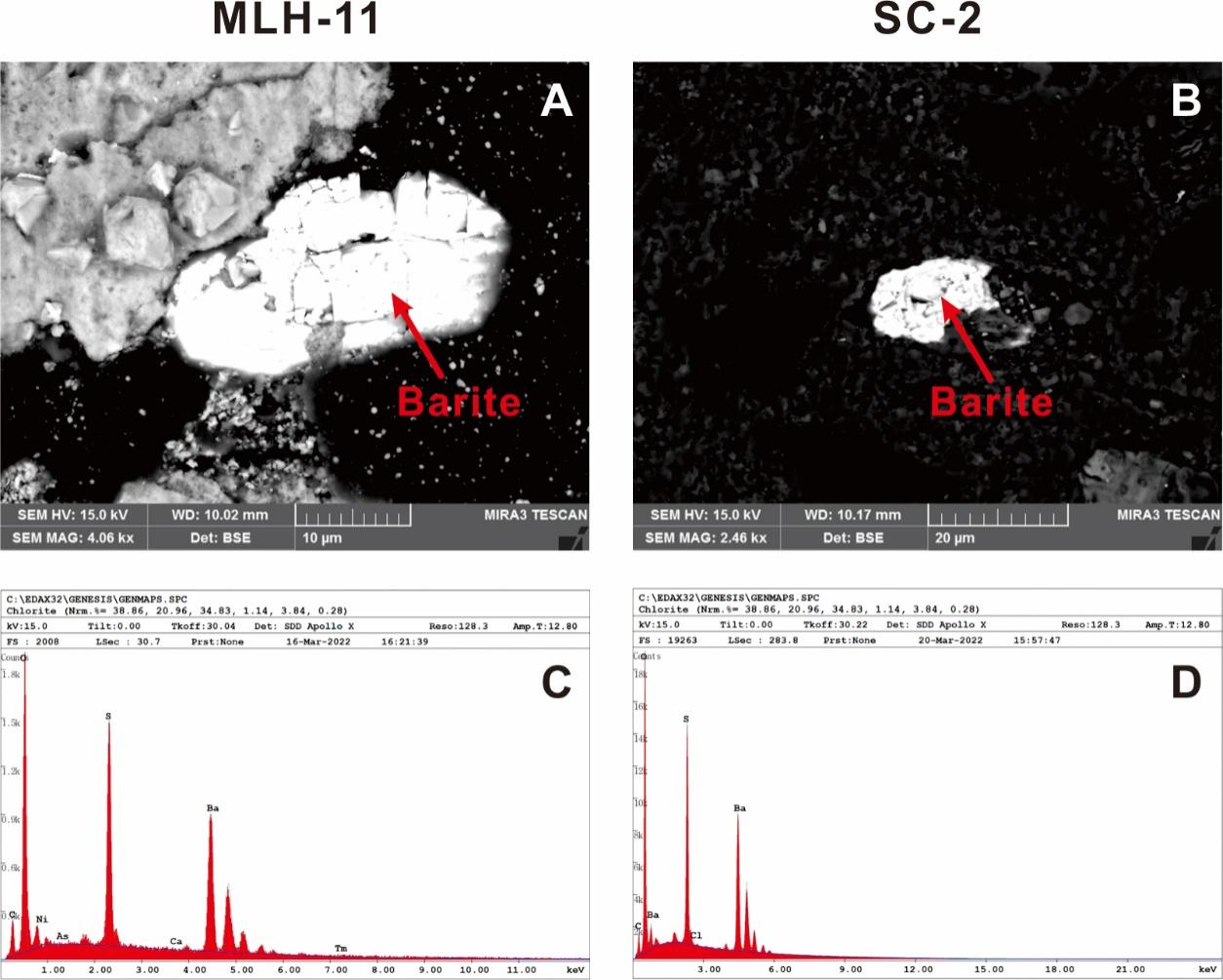


Fig. S4.

**SEM and EDS results.** SEM photographs of barite particles in the representative metalliferous black shales from (A) Maluhe and (B) Sancha. EDS results with signal peaks of O, S, and Ba, validating the presence of barites in the selected samples from (C) Maluhe and (D) Sancha.


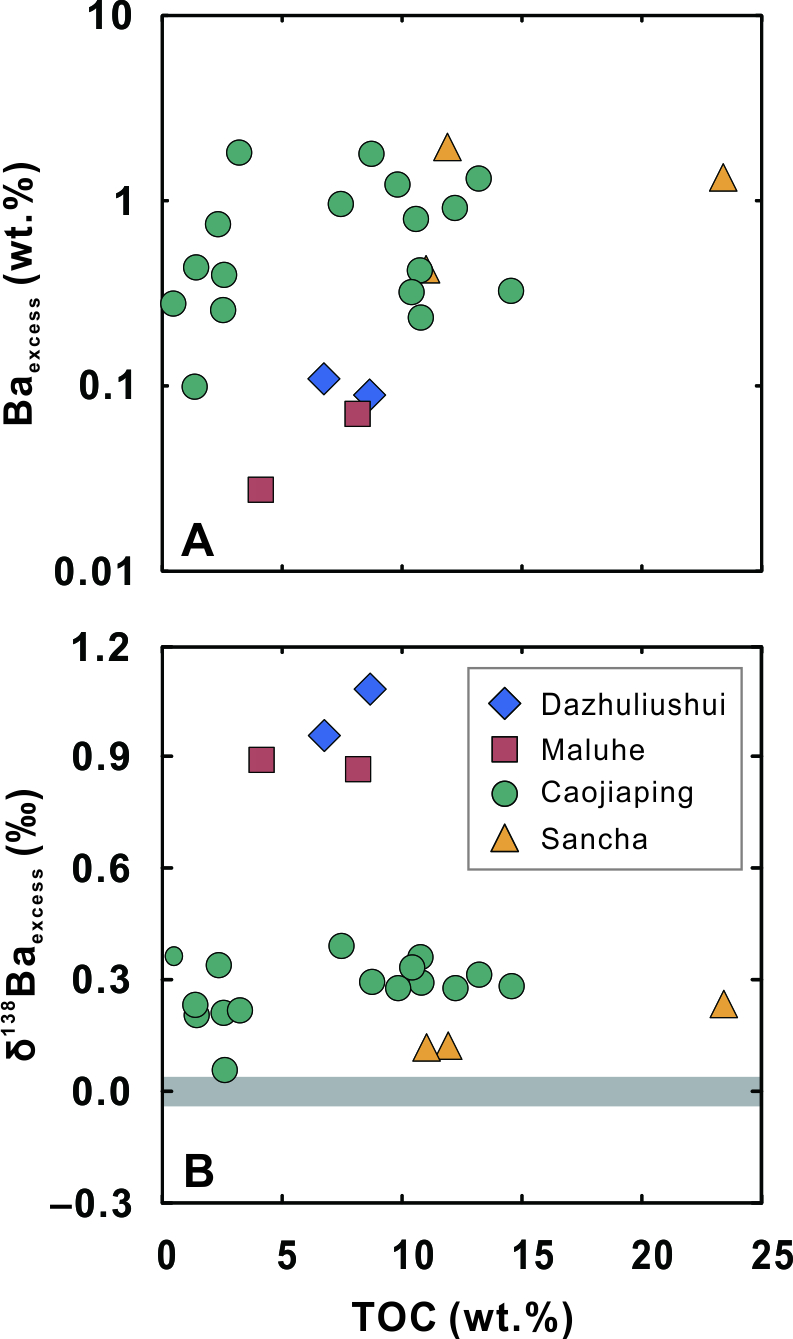


Fig. S5.

**Geochemical cross-plots.** TOC contents versus (A) Ba_excess_ contents and (B) δ^138^Ba_excess_ values for metalliferous black shales of the lower Niutitang Formation at Dazhuliushui, Maluhe, Caojiaping, and Sancha. In (B), the grey horizontal bar represents the average δ^138^Ba value of the upper continental crust (0.00 ± 0.04‰) [65].

Table S1.

Sample information, contents of Al, Ba, and TOC, δ^138^Ba values, *f*_excess_, Ba_excess_ contents, and δ^138^Ba_excess_ values for metalliferous black shales of the lower Niutitang Formation at Dazhuliushui, Maluhe, Caojiaping, and Sancha. The contents of major elements, trace elements, and TOC are cited from Xu *et al.* [21,22] and Lehmann *et al.* [20]. The δ^138^Ba values of SGR-1 and BHVO-2 are from Nan *et al.* [65] and An *et al.* [84], respectively.

Table S2.

Evolutions in mean marine sulfate and Ba concentrations from 565 Ma to 480 Ma. Sulfate concentration data are from Algeo *et al.* [47].

References

1. Gong Y, Zeng Z and Zhou C *et al.* Barium isotopic fractionation in latosol developed from strongly weathered basalt. *Sci Total Environ* 2019; **687**: 1295–1304.
2. Gou LF, Jin ZD and Galy A *et al.* Seasonal riverine barium isotopic variation in the middle Yellow River: Sources and fractionation. *Earth Planet Sci Lett* 2020; **531**: 115990.
3. Cao Z, Siebert C and Hathorne EC *et al.* Corrigendum to “Constraining the oceanic barium cycle with stable barium isotopes” [Earth Planet. Sci. Lett. 434 (2016) 1–9]. *Earth Planet Sci Lett* 2020; **530**: 116003.
4. Mayfield KK, Eisenhauer A and Ramos DPS *et al.* Groundwater discharge impacts marine isotope budgets of Li, Mg, Ca, Sr, and Ba. *Nat Commun* 2021; **12**: 148.
5. Edmond JM, Measures C and McDuff RE *et al.* Ridge crest hydrothermal activity and the balances of the major and minor elements in the ocean: The Galapagos data. *Earth Planet Sci Lett* 1979; **46**: 1–18.
6. Horner TJ, Kinsley CW and Nielsen SG. Barium-isotopic fractionation in seawater mediated by barite cycling and oceanic circulation. *Earth Planet Sci Lett* 2015. **430**: 511–522.
7. Cao Z, Siebert C and Hathorne EC *et al.* Constraining the oceanic barium cycle with stable barium isotopes. *Earth Planet Sci Lett* 2016; **434**: 1–9.
8. Bates SL, Hendry KR and Pryer HV *et al.* Barium isotopes reveal role of ocean circulation on barium cycling in the Atlantic. *Geochim Cosmochim Acta* 2017; **204**: 286–299.
9. Hsieh YT and Henderson GM. Barium stable isotopes in the global ocean: Tracer of Ba inputs and utilization. *Earth Planet Sci Lett* 2017; **473**: 269–278.
10. Martinez-Ruiz F, Jroundi F and Paytan A *et al.* Barium bioaccumulation by bacterial biofilms and implications for Ba cycling and use of Ba proxies. *Nat Commun* 2018; **9**: 1969.
11. Martinez-Ruiz F, Paytan A and Gonzalez-Munoz MT *et al.* Barite formation in the ocean: Origin of amorphous and crystalline precipitates. *Chem Geol* 2019; **511**: 441–451.
12. Monnin C, Jeandel C and Cattaldo T *et al.* The marine barite saturation state of the world's oceans. *Mar Chem* 1999; **65**: 253–261.
13. Kravchenko J, Darrah TH and Miller RK et al. A review of the health impacts of barium from natural and anthropogenic exposure. Environ Geochem Health 2014; 36: 797–814.
14. Moens T and Vincx M. Temperature and salinity constraints on the life cycle of two brackish-water nematode species. *J Exp Mar Biol Ecol* 2000; **243**: 115–135.
15. Schoepfer SD, Shen J and Wei H *et al.* Total organic carbon, organic phosphorus, and biogenic barium fluxes as proxies for paleomarine productivity. *Earth-Sci Rev* 2015; **149**: 23–52.
16. Pretet C, van Zuilen K and Nägler TF *et al.* Constraints on barium isotope fractionation during aragonite precipitation by corals. *Depos Rec* 2016; **1**: 118–129.
17. Hemsing F, Hsieh YT and Bridgestock L *et al.* Barium isotopes in cold-water corals. *Earth Planet Sci Lett* 2018; **491**: 183–192.
18. Cao ZM, Siebert C and Hathorne EC *et al.* Constraining barium isotope fractionation in the upper water column of the South China Sea. *Geochim Cosmochim Acta* 2020; **288**: 120–137.
19. An YJ, Li X and Zhang ZF. Barium isotopic compositions in thirty-four geological reference materials analysed by MC-ICP-MS. *Geostand Geoanal Res* 2020; **44**: 183–199.
